# Two compound heterozygous variants in the *CLN8* gene are responsible for neuronal cereidolipofuscinoses disorder in a child: a case report

**DOI:** 10.3389/fped.2024.1379254

**Published:** 2024-05-01

**Authors:** Federico Baltar, Camila Simoes, Francisco Garagorry, Martín Graña, Soledad Rodríguez, María Haydée Aunchayna, Alejandra Tapié, Alfredo Cerisola, Gabriel González, Hugo Naya, Lucía Spangenberg, Víctor Raggio

**Affiliations:** ^1^ Unidad Académica de Neuropediatría, Facultad de Medicina, Universidad de la República, Montevideo, Uruguay; ^2^ Departamento de Genética, Facultad de Medicina, Universidad de la República, Montevideo, Uruguay; ^3^ Unidad de Bioinformática, Institut Pasteur de Montevideo, Montevideo, Uruguay; ^4^ Departamento Básico de Medicina, Hospital de Clínicas, Facultad de Medicina, Universidad de la República, Montevideo, Uruguay; ^5^ Unidad Académica de Anatomía Patológica, Hospital de Clínicas, Facultad de Medicina Universidad de la República, Montevideo, Uruguay; ^6^ Departamento de Producción Animal y Pasturas, Facultad de Agronomía, Universidad de la República, Montevideo, Uruguay

**Keywords:** genomics, whole genome sequencing, neurological disorders, neurodegeneration, *CLN8*, ceroid lipofuscinosis

## Abstract

**Background:**

Neuronal Ceroid Lipofuscinosis (NCL) disorders, recognized as the primary cause of childhood dementia globally, constitute a spectrum of genetic abnormalities. CLN8, a subtype within NCL, is characterized by cognitive decline, motor impairment, and visual deterioration. This study focuses on an atypical case with congenital onset and a remarkably slow disease progression.

**Methods:**

Whole-genome sequencing at 30× coverage was employed as part of a national genomics program to investigate the genetic underpinnings of rare diseases. This genomic approach aimed to challenge established classifications (vLINCL and EPMR) and explore the presence of a continuous phenotypic spectrum associated with *CLN8*.

**Results:**

The whole-genome sequencing revealed two novel likely pathogenic mutations in the *CLN8* gene on chromosome 8p23.3. These mutations were not previously associated with CLN8-related NCL. Contrary to established classifications (vLINCL and EPMR), our findings suggest a continuous phenotypic spectrum associated with CLN8. Pathological subcellular markers further validated the genomic insights.

**Discussion:**

The identification of two previously undescribed likely pathogenic *CLN8* gene mutations challenges traditional classifications and highlights a more nuanced phenotypic spectrum associated with CLN8. Our findings underscore the significance of genetic modifiers and interactions with unrelated genes in shaping variable phenotypic outcomes. The inclusion of pathological subcellular markers further strengthens the validity of our genomic insights. This research enhances our understanding of *CLN8* disorders, emphasizing the need for comprehensive genomic analyses to elucidate the complexity of phenotypic presentations and guide tailored therapeutic strategies. The identification of new likely pathogenic mutations underscores the dynamic nature of *CLN8*-related NCL and the importance of individualized approaches to patient management.

## 1 Introduction

Neuronal Ceroid Lipofuscinosis (NCL) disorders are the most common neurodegenerative diseases in childhood, and are reported as the leading cause of childhood dementia worldwide (1, 2). The higher prevalence of selected forms of NCL in restricted geographic areas is historical and might reflect early progress in molecular diagnosis in some countries (3). Epidemiological data indicates an incidence of about 1/1,000,000 (4), and the estimated total incidence ranges from 0.01 to 9 per 100 000 live births (5, 6).

Ceroid-lipofuscinosis, neuronal 8 (CLN8) belongs to the NCL disorders and predominantly affects the central nervous system, leading to progressive cognitive decline, motor impairment, and visual deterioration. Initial descriptions of CLN8 delineated two clearly distinct phenotypes. The late infantile Turkish variant (vLINCL) represents the most severe form, typically initiating between 2 and 7 years (7, 8). Affected patients develop myoclonic epilepsy and ataxia, accompanied by developmental regression leading to the loss of the ability to walk and talk. Cognitive capacity progressively declines in this form and affected individuals rarely survive beyond late childhood or early adolescence (7). Northern epilepsy or progressive epilepsy with mental retardation (EPMR) is the less severe form of the disease, characterized by recurrent seizures (9). It usually does not present myoclonus or visual failure, unlike vLINCL. The usual onset occurs between 5 and 10 years. As the disease advances, affected individuals develop ataxia, other motor dysfunctions, and a gradual decline in cognitive abilities (9, 10). EPMR has a much slower course, and patients usually live longer than those with vLINCL (11).

The genetic basis of CLN8 involves loss-of-function mutations in the *CLN8* gene, located on chromosome 8p23.3, which encodes a transmembrane endoplasmic reticulum protein. The function of the CLN8 protein has yet to be entirely elucidated, but it is required for the endoplasmic reticulum-to-Golgi transfer of lysosomal enzymes. CLN8 deficiency leads to depletion of soluble enzymes in the lysosome, thus impairing lysosome biogenesis and leading to a lysosomal storage disorder (12). It has been demonstrated that CLN8 protein forms a complex with the product of *CLN6*, another gene whose loss of function is associated with NCL, necessary to recruit lysosomal enzymes and promote their Golgi transfer (13). Apparently, the knockdown of CLN8 led to an increase in the size of the Golgi apparatus, the number of mobile vesicles, and the velocity of endo-lysosomes, alongside significant lysosomal alkalinization in CLN8-deficient cells (14). Also, these findings (14) indicate that *CLN8* deficiency is involved in atrophy, shortening, and degeneration of the neural dendritic tree. These suggest that the abnormalities induced by *CLN8* deficiency in the basal endo-lysosomal system underlie morphological changes in neurons that ultimately contribute to the characteristic neurodegeneration observed in this NCL.

Despite classically defined age windows, an increasing number of patients demonstrate variable progression and onset age, even within the same family (15–17). This clinical variation is typically attributed to patients' genetic background [i.e., modifier genes (15)] and the severity of causal mutations. Recently, mutations in unrelated genes have been considered modifiers of gene expression, and interactions between mutated genes and modifiers can lead to clinical variations and observed phenotypic heterogeneity (1). Rare cases with *CLN8* pathogenic variants report congenital presentations or symptoms onset in the first year of life, as well as presentations deviating from previous NCL paradigms, such as the absence of myoclonic seizures or visual sensory loss (10, 18, 19). These reports further suggest a continuous spectrum of phenotypes, a phenotypic heterogeneity associated with CLN8 instead of a clear distinction between EPMR and vLINCL (20, 21).

Here, we present a congenital case of *CLN8* with a very slow disease progression confirmed by pathologic subcellular markers. Whole genome sequencing (WGS) revealed the diagnosis in the context of a national genomics academic program.

## 2 Case report

The patient is a ten-year-old Uruguayan male, son of non-consanguineous parents, with no relevant family or perinatal history. In the neonatal stage, he presented sucking disorders, which led to malnutrition in the first trimester of life. He presented developmental compromise from an early age, achieving cephalic support at nine months and independent standing at three years of age, without ever having acquired independent walking or oral language. He never presented a loss of acquired maturational behaviors. At ten years of age, he presents a severe intellectual deficit and autism spectrum disorder (ASD), characterized by limited communicative intention and visual contact with frequent manual stereotypies (flapping).

At three years of life, he started with epilepsy in the form of asymmetric and alternating hemibody focal tonic seizures, occasionally evolving to generalized clonic seizures, always of short duration. He never presented with myoclonic seizures. Since the onset of seizures, he has always presented several seizures per week, with a poor response to multiple antiepileptic drugs (valproic acid, levetiracetam, phenobarbital, clobazam, cannabis).

Physical examination showed a normal head circumference without dysmorphic features, poor eye contact, and frequent manual stereotypies. He presented nonparetic hypotonia with normal osteotendinous reflexes and a plantar cutaneous reflex in bilateral flexion.

**Figure 1:**
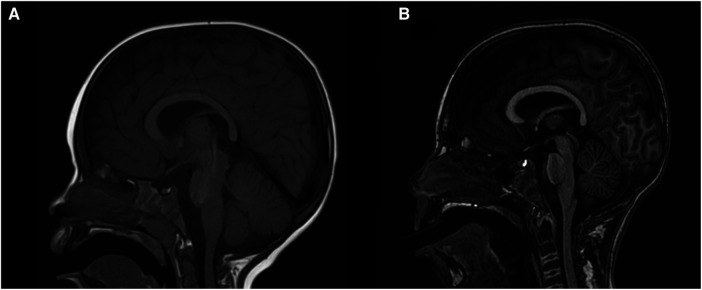
Brain MRI. (**A**) Sagittal T1 sequences at two years old, and (**B**) nine years old. Progressive cerebellar and discrete cerebral atrophy are observed.

A first brain magnetic resonance imaging (MRI) was performed at two years of age, which was normal. An additional brain MRI was done at nine years of age, which showed cerebellar and mild cerebral atrophy (Figure 1). A basic metabolic study was performed, which included normal urine organic acids and normal blood amino acids. Transferrin isoelectrophoresis was normal. Visual and auditory evoked potential tests at the age of 3 years were normal. Multiple electroencephalograms (EEG) have been performed since age three, showing a poorly organized and slow background rhythm with focal activity in different topographies. No EEG with low-frequency photic stimulation was conducted.

Karyotyping and array comparative genomic hybridization were normal.

He did not receive any other pharmacological treatments. Since infancy, he has been receiving speech and occupational therapy, with limited progress in cognitive domains. The child attends a particular education school.

Although the first clinical manifestations occurred congenitally and the child has achieved few developmental milestones throughout his life, there was no progression of the condition beyond the appearance of his epilepsy, behaving almost statically.

## 3 Methods

### 3.1 Standard protocol approvals and patient consents

This project was approved by the Ethics Committee from the Institut Pasteur de Montevideo (IP011-17/CEI/LC/MB). Written informed consent was obtained from the patient's guardians.

### 3.2 Whole genome sequencing and bioinformatics analysis

We carried out the WGS of the patient with paired-end reads protocol on a Hiseq X ten Illumina sequencer (30x, 150PE), with an average depth of ∼70×. The quality of reads was analyzed using FastQC (http://www.bioinformatics.babraham.ac.uk/projects/fastqc/), and they were mapped onto the human reference genome (GRCh37) using BWA (22). Only unique reads mapping in proper pairs were further considered. Variant calling was performed using GATK (best practices) (23), and ANNOVAR (24) was used for annotation. The mitochondrial genome was also analyzed using MToolBox for mapping, haplogroup prediction, variant calling and annotation, and heteroplasmy estimation. More details are in the Supplementary Material.

Sanger sequencing was used to confirm the mutations in the index case.

### 3.3 Electron microscopy

Microscopic analysis of ultrastructural patterns of cellular deposits helps categorize patients into possible NCL subtypes, as lipopigment morphotypes generally strongly correlate with genotype (2). Skin biopsy (punch) was performed and fixed in a mixture of 2.5% glutaraldehyde, 2% formaldehyde, 0.1 M sodium cacodylate buffer, pH 7.4, 2 hs at room temperature. More details are in the Supplementary Material.

## 4 Results

### 4.1 Two compound heterozygous variants are likely causative of the patient's phenotype

WGS delivered 785.952.690 paired reads that passed the QC controls. 753.615.267 reads (95.89%) were mapped onto the reference genome (GRCh37), and 729.372.752 reads (92.80%) were properly paired.

**Figure 2:**
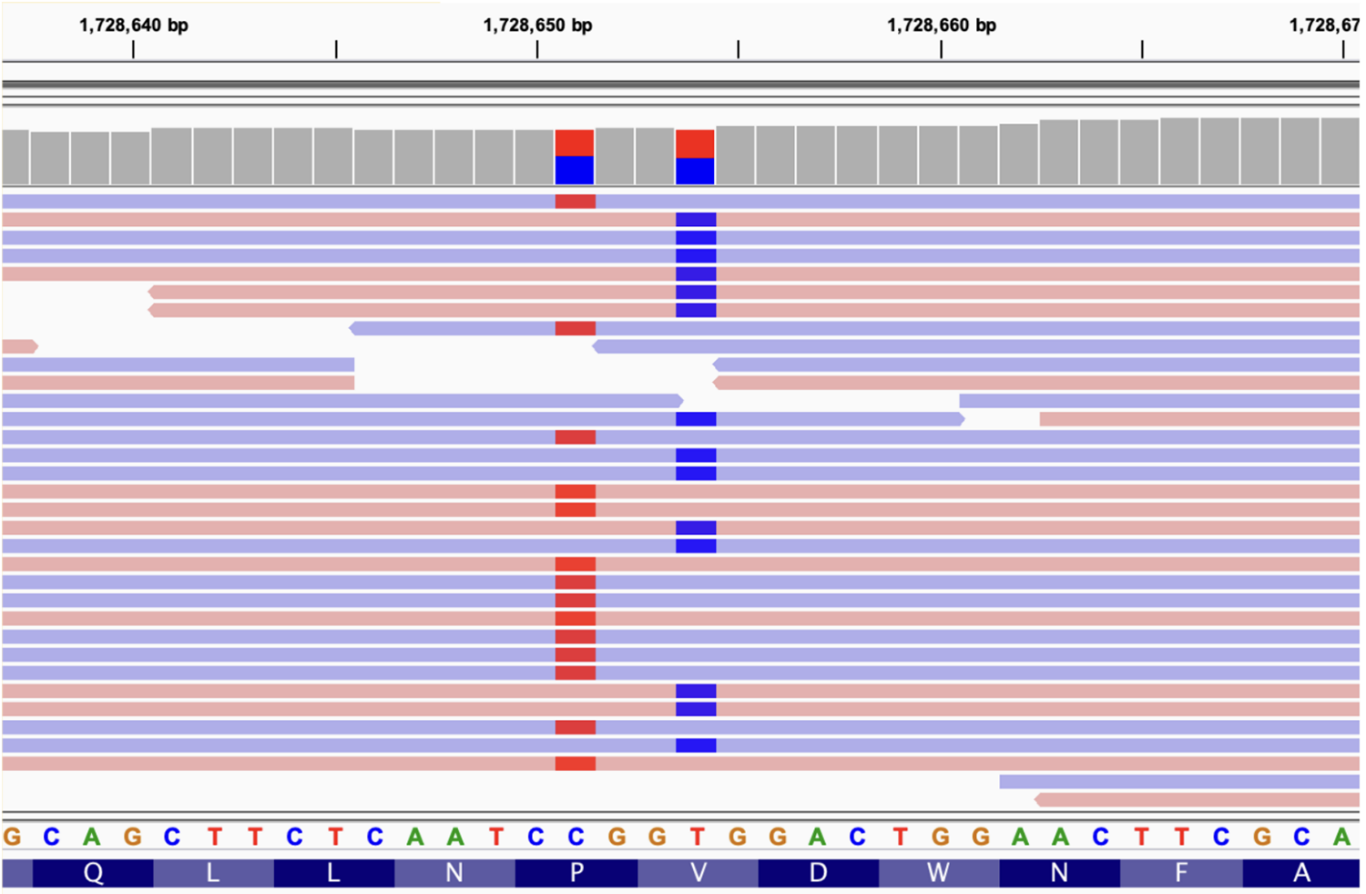
IGV view of the reads mapping onto the *CLN8* gene (partial view). The two compound heterozygous variants are located (1728651 and 1728654). Blue and red bars mark the presence of a single nucleotide variant allele in the reads (light blue and red horizontal bars). Not all reads are shown (the coverage for the two variants is 35x). Reference genomic and protein sequences are shown below. The two variants are always in different reads, being aligned to different chromosomes (compound heterozygosity).

The data showed two variants *in trans* within the *CLN8* gene (Figure 2). One allele harbors the variant chr8:1728651, C/T (NM_018941:exon3:c.C779T:p.Pro260Leu), which has been reported in heterozygosity in 1000G, ExAC and gnomAD databases in 6, 31 and 78 individuals, respectively, resulting in allele frequencies of 0.00119808, 0.0003 and 0.0007, respectively (Figure 2, left variant). Several physico-chemicals *in silico* scores classified this variant as pathogenic (PolyPhen (25), SIFT (26), MutationTaster (27), FATHMM (28)), and it has a CADD (29) Phred meta predictor value of 21. Conservation scores were all high (LRT (30), GERP (31), phyloP (32)).

The other allele harbors the variant chr8:1728654, T/C (NM_018941:exon3:c.T782C:p.Val261Ala) that was previously reported in heterozygosity in only three individuals in the gnomAD database with a population frequency of 0.00001193 (Figure 2, right variant). *In silico* scores also classify this variant as deleterious (PolyPhen, Sift, MutationTaster, FATHMM) and its CADD score is 15.65. Additionally, this Val261Ala variant alters the first amino acid of the signal peptide 261-VDWNF-265 of *CLN8* protein, which is necessary for its transport from the endoplasmic reticulum to the Golgi apparatus (12). The Pro260Leu variant, referred to as first, alters the amino acid immediately upstream of this signal sequence since the two variants are in adjacent codons.

Both mutations have been submitted to ClinVar under accession numbers SCV004697978 (February 29th, 2024) and SCV004697994 (March 4th, 2024), respectively.

The two variants found can be classified as likely pathogenic according to ACMG criteria: i. Pro260Leu missense variant has low frequency (Pathogenic Moderate rule 2; PM2), detected *in trans* with another likely pathogenic mutation (PM3), is a missense variant in a gene where missense variants are a common mechanism of disease (Supporting pathogenic rule 2; PP2) (8). Additionally, multiple lines of evidence of *in silico* scores support a deleterious effect (PP3); the patient's phenotype is highly specific for a disease with a single gene etiology (PP4). Integrating all rules, 2 PM and 3 PP, leads to a likely pathogenic classification. This variant has been reported in Clinvar with conflicting interpretations of pathogenicity, but mostly as a variant of uncertain significance (https://www.ncbi.nlm.nih.gov/clinvar/variation/205196/); ii. Val261Ala missense variant can also be classified as likely pathogenic with the same rules applied. This variant has been reported in Clinvar in a few patients and is always classified as a variant of uncertain significance (https://www.ncbi.nlm.nih.gov/clinvar/variation/1000047/). We have identified the two variants in compound heterozygosity with additional data to consider them as likely pathogenic mutations.

### 4.2 Electron microscopy

**Figure 3:**
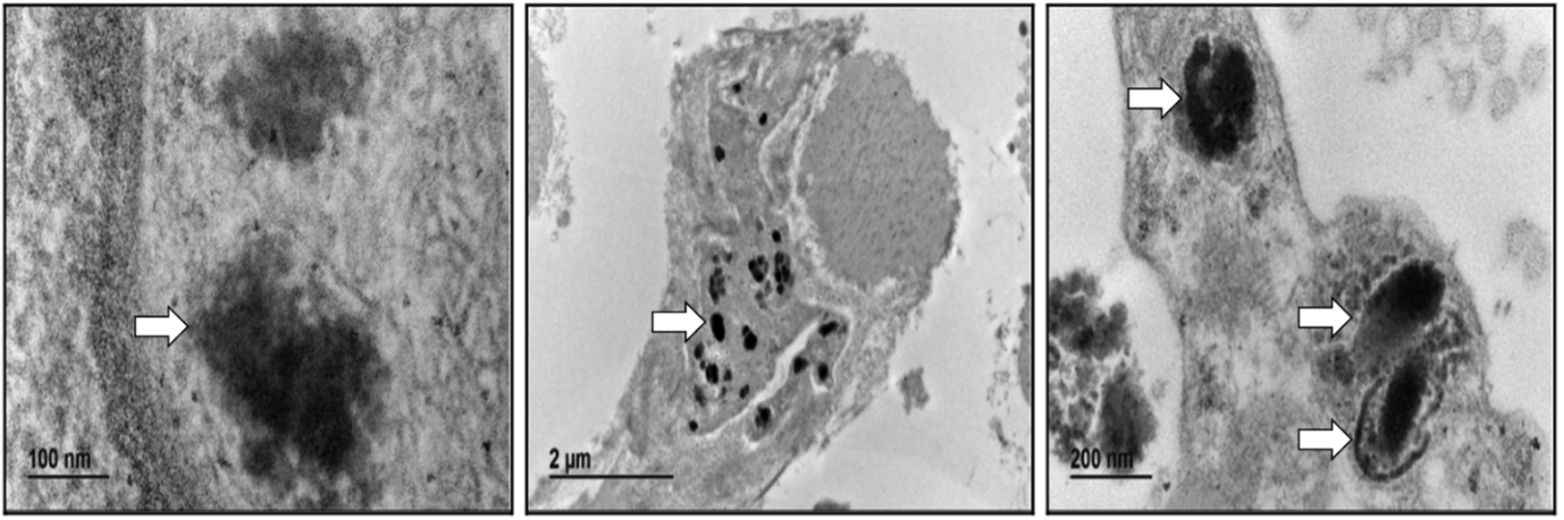
Electron microscopy. Abnormal intracellular deposits were observed in the skin's hypodermal fibroblast, consistent with the diagnosis of GRODs.

Skin biopsy analysis has become the most common pathological diagnostic tool for NCL and a range of other childhood neurodegenerative diseases, where abnormal accumulation of macromolecular material is a prominent feature and a pathogenic hallmark (33). In CLN8-related cases, electron microscopy usually reveals storage material adopting curved profiles attached to the membrane, fingerprints, and limited deposition areas of osmiophilic granular material (34, 35). In the present patient, only granular osmiophilic deposits (GROD) were observed (Figure 3) despite performing additional deeper sections.

## 5 Discussion

We present a new case of NCL caused by two previously unreported likely pathogenic mutations in the *CLN8* gene. The patient had an atypical presentation, being congenital but with a very slow progression of the disease, an observation that supports a relatively broad spectrum of presentation of NCL types.

Epilepsy is common to all forms of NCL (1). While generalized epileptic seizures [Generalized tonic-clonic seizure (GTCS), myoclonic, atonic, or absence] are present in all known cases of CLN8, focal seizures, as reported in our case, have been infrequently documented. It is also noteworthy that spontaneous myoclonus was absent in our case. Myoclonus is a typical feature of NCL, although there are reports of cases lacking this type of crisis. As observed in our case, the EEG shows a progressive slowing of the background rhythm, along with interictal discharges, including slow spike-wave or polyspike-wave complexes (20). A characteristic feature of the EEG in NCL patients is the paroxysmal response to spike-wave, evoked by intermittent low-frequency photic stimulation (1–3 Hz) (1, 36). However, different types of NCL show varying susceptibility to photoparoxysmal response (2). Unfortunately, performing a low-frequency photic stimulation EEG in our case was impossible.

The progression and severity of deterioration vary widely and generally parallel to the overall course of the disease (20). In the present case, it has been congenital and very slowly progressive, simulating a non-progressive course. Behavioral phenotypes with ASD features have also been reported before (2, 36). Heterozygous variations in *CLN8* have been proposed to confer increased susceptibility to ASD (37).

Brain MRI may appear normal in the early stages of the disease. Cerebellar atrophy has become a consistent feature over time and is present in our patient at age 9, affecting both the vermis and cerebellar hemispheres as previously described (2, 36). Supratentorial cerebral atrophy is often observed but could be a late feature, as in our patient (20). A relevant aspect of this clinical case is two MRIs separated seven years from each other, in which progressive brain atrophy is not observed. As we already mentioned, we did not observe neurological clinical progressivity either.

Treatment is symptomatic and multidisciplinary, focusing on providing the best quality of life (1, 38). Some pharmacological treatments are in preclinical studies: AMPA receptor modulator therapy (ZK-187638), retigabine, insulin-like growth factor 1, as well as gene therapy (vectors-Amicus Therapeutics' AAV9-CLN8) (11, 16). Stem cell therapy in mouse and dog models of CLN8 led to *CLN8* expression in the spinal cord but not in the CNS (11). Most of these therapies in development are potentially disease-modifying, meaning they may delay or even halt disease progression, but few are likely to reverse the disease, even partially. Therefore, early diagnosis and treatment will become increasingly crucial as these damage-limiting interventions become available (16). Reports on life expectancy and mortality in NCL are scarce. Variability within each form, even in *CLN8*, does not allow precise life expectancy predictions in individual patients. Over the last two decades, generally longer survival has been observed in patients with many forms of NCL, regardless of mutation severity. Such findings can largely be attributed to improved care for these patients (1).

Ultrastructural examination through skin biopsy remains useful for confirming genetically undiagnosed atypical forms (2). In this case, it was motivated by the variants detected in the *CLN8* gene after WGS analysis. Results showed typical findings, consisting of abnormal accumulation of macromolecular material.

To date, ClinVar reports eight missense, 14 frameshifts, 21 nonsense, and one splice site *CLN8* mutations classified as pathogenic (https://www.ncbi.nlm.nih.gov/clinvar/, accessed on 26th February 2024). Studies have also been published reporting those mutations associated specifically with *CLN8* disorder (accessible through the NCL resource https://www.ucl.ac.uk/ncl-disease/). According to the numbers obtained from ClinVar, missense mutations appear to be an unusual or under-detected cause in CLN8-related disorders.

A close inspection of the substitutions also supports pathogenicity considerations of the patient's variants. First, Pro260 is a strictly conserved residue, probably due to a stabilizing role of the C-terminus of an alpha helix in its cytoplasmic face, just after the transmembrane segment (39–41). The Pro260Leu substitution may imply a conformational distortion of the protein, affecting its dimerization (42) and hence its downstream actions, which include exporting protein products to the lysosome (12). Second, the effect of the Val261Ala mutation may be less dramatic, yet changing the start of the 261VDWNF265 motif, an export signal from the endoplasmic reticulum to the Golgi (43). In line with a recessive inheritance pattern and the fact that the parents are healthy, we surmise that both variants compromise the protein function. Cascading effects at the molecular and cellular level would result from expressed pools of proteins carrying one or the other mutation.

The discovery of these two missense variants in a new case of NCL expands the repertoire of reported variants associated with this disorder, providing insights into the intricate nature of phenotypic presentations in *CLN8*. Functional studies providing evidence of a loss of function effect of the two variants are needed, and the impact of specific missense mutations in the product of *CLN8* deserves further investigation. Defining genetic (and eventually epigenetic) variation that impacts *CLN8* function will be essential to understanding the increasingly complex relationships between NCL genotype and phenotype and advancing treatment options (15).

## Data Availability

The datasets presented in this study can be found in online repositories. The names of the repository/repositories and accession number(s) can be found in the article.

## References

[1] Simonati A Williams RE. Neuronal ceroid lipofuscinosis: the multifaceted approach to the clinical issues, an overview. Front. Neurol. (2022) 13:811686. 10.3389/fneur.2022.811686 35359645 PMC8961688

[2] Kaminiów K Kozak S Paprocka J. Recent insight into the genetic basis, clinical features, and diagnostic methods for neuronal ceroid lipofuscinosis. Int J Mol Sci. (2022) 23(10):5729. 10.3390/ijms23105729 35628533 PMC9145894

[3] Guelbert G Venier AC Cismondi IA Becerra A Vazquez JC Fernández EA Neuronal ceroid lipofuscinosis in the South American-Caribbean Region: an epidemiological overview. Front Neurol. (2022) 13:920421. 10.3389/fneur.2022.920421 36034292 PMC9412946

[4] Haltia M Goebel HH. The neuronal ceroid-lipofuscinoses: a historical introduction. Biochim Biophys Acta. (2013) 1832:1795–800. 10.1016/j.bbadis.2012.08.012 22959893

[5] Santorelli FM Garavaglia B Cardona F Nardocci N Bernardina BD Sartori S Molecular epidemiology of childhood neuronal ceroid-lipofuscinosis in Italy. Orphanet J Rare Dis. (2013) 8:19. 10.1186/1750-1172-8-19 23374165 PMC3570295

[6] Moore SJ Buckley DJ MacMillan A Marshall HD Steele L Ray PN The clinical and genetic epidemiology of neuronal ceroid lipofuscinosis in Newfoundland. Clin Genet. (2008) 74:213–22. 10.1111/j.1399-0004.2008.01054.x 18684116

[7] Mitchell WA Wheeler RB Sharp JD Bate SL Gardiner RM Ranta US Turkish variant late infantile neuronal ceroid lipofuscinosis (CLN7) may be allelic to CLN8. Eur J Paediatr Neurol. (2001) 5 Suppl A:21–7. 10.1053/ejpn.2000.0429 11589000

[8] Ranta S Topcu M Tegelberg S Tan H Ustübütün A Saatci I Variant late infantile neuronal ceroid lipofuscinosis in a subset of Turkish patients is allelic to northern epilepsy. Hum Mutat. (2004) 23:300–5. 10.1002/humu.20018 15024724

[9] Ranta S Lehesjoki AE. Northern epilepsy, a new member of the NCL family. Neurol Sci. (2000) 21:S43–7. 10.1007/s100720070039 11073227

[10] Sahin Y Güngör O Gormez Z Demirci H Ergüner B Güngör G Exome sequencing identifies a novel homozygous CLN8 mutation in a Turkish family with northern epilepsy. Acta Neurol Belg. (2017) 117:159–67. 10.1007/s13760-016-0721-3 27844444

[11] Rosenberg JB Chen A Kaminsky SM Crystal RG Sondhi D. Advances in the treatment of neuronal ceroid lipofuscinosis. Expert Opin Orphan Drugs. (2019) 7:473–500. 10.1080/21678707.2019.1684258 33365208 PMC7755158

[12] Sifers RN Santorelli FM Sardiello M. CLN8 Is an endoplasmic reticulum cargo receptor that regulates lysosome biogenesis. Nat Cell Biol. (2018) 20:1370–7. 10.1038/s41556-018-0228-7 30397314 PMC6277210

[13] Bajaj L Sharma J di Ronza A Zhang P Eblimit A Pal R A CLN6-CLN8 complex recruits lysosomal enzymes at the ER for Golgi transfer. J Clin Invest. (2020) 130:4118–32. 10.1172/JCI130955 32597833 PMC7410054

[14] Pesaola F Quassollo G Venier AC De Paul AL Noher I Bisbal M. The neuronal ceroid lipofuscinosis-related protein CLN8 regulates endo-lysosomal dynamics and dendritic morphology. Biol Cell. (2021) 113:419–37. 10.1111/boc.202000016 34021618

[15] Butz ES Chandrachud U Mole SE Cotman SL. Moving towards a new era of genomics in the neuronal ceroid lipofuscinoses. Biochim Biophys Acta Mol Basis Dis. (2020) 1866:165571. 10.1016/j.bbadis.2019.165571 31678159

[16] Specchio N Ferretti A Trivisano M Pietrafusa N Pepi C Calabrese C Neuronal ceroid lipofuscinosis: potential for targeted therapy. Drugs. (2021) 81:101–23. 10.1007/s40265-020-01440-7 33242182

[17] Mahajnah M Zelnik N. Phenotypic heterogeneity in consanguineous patients with a common CLN8 mutation. Pediatr Neurol. (2012) 47:303–5. 10.1016/j.pediatrneurol.2012.05.016 22964447

[18] Pesaola F Kohan R Cismondi IA Guelbert N Pons P Oller-Ramirez AM Congenital CLN8 disease of neuronal ceroid lipofuscinosis: a novel phenotype. Rev Neurol. (2019) 68:155–9. 10.33588/rn.6804.2018217 30741402

[19] Striano P Specchio N Biancheri R Cannelli N Simonati A Cassandrini D Clinical and electrophysiological features of epilepsy in Italian patients with CLN8 mutations. Epilepsy Behav. (2007) 10:187–91. 10.1016/j.yebeh.2006.10.009 17129765

[20] Badura-Stronka M Winczewska-Wiktor A Pietrzak A Hirschfeld AS Zemojtel T Wołyńska K CLN8 Mutations presenting with a phenotypic continuum of neuronal ceroid lipofuscinosis-literature review and case report. Genes (Basel). (2021) 12:956. 10.3390/genes12070956 34201538 PMC8307369

[21] Sharkia R Zalan A Zahalka H Kessel A Asaly A Al-Shareef W CLN8 gene compound heterozygous variants: a new case and protein bioinformatics analyses. Genes (Basel). (2022) 13(8):1393. 10.3390/genes13081393 36011304 PMC9407845

[22] Li H Durbin R. Fast and accurate short read alignment with burrows–wheeler transform. Bioinformatics. (2009) 25:1754–60. 10.1093/bioinformatics/btp324 19451168 PMC2705234

[23] McKenna A Hanna M Banks E Sivachenko A Cibulskis K Kernytsky A The genome analysis toolkit: a MapReduce framework for analyzing next-generation DNA sequencing data. Genome Res. (2010) 20:1297–303. 10.1101/gr.107524.110 20644199 PMC2928508

[24] Wang K Li M Hakonarson H. ANNOVAR: functional annotation of genetic variants from high-throughput sequencing data. Nucleic Acids Res. (2010) 38:e164. 10.1093/nar/gkq603 20601685 PMC2938201

[25] Adzhubei IA Schmidt S Peshkin L Ramensky VE Gerasimova A Bork P A method and server for predicting damaging missense mutations. Nat Methods. (2010) 7:248–9. 10.1038/nmeth0410-248 20354512 PMC2855889

[26] Ng PC Henikoff S. Predicting deleterious amino acid substitutions. Genome Res. (2001) 11:863–74. 10.1101/gr.176601 11337480 PMC311071

[27] Schwarz JM Cooper DN Schuelke M Seelow D. Mutationtaster2: mutation prediction for the deep-sequencing age. Nat Methods. (2014) 11:361–2. 10.1038/nmeth.2890 24681721

[28] Shihab HA Gough J Cooper DN Stenson PD Barker GLA Edwards KJ Predicting the functional, molecular, and phenotypic consequences of amino acid substitutions using hidden markov models. Hum Mutat. (2013) 34:57–65. 10.1002/humu.22225 23033316 PMC3558800

[29] Kircher M Witten DM Jain P O'Roak BJ Cooper GM Shendure J. A general framework for estimating the relative pathogenicity of human genetic variants. Nat Genet. (2014) 46:310–5. 10.1038/ng.2892 24487276 PMC3992975

[30] Chun S Fay JC. Identification of deleterious mutations within three human genomes. Genome Res. (2009) 19:1553–61. 10.1101/gr.092619.109 19602639 PMC2752137

[31] Cooper GM Stone EA Asimenos G NISC Comparative Sequencing Program Green ED Batzoglou S Distribution and intensity of constraint in mammalian genomic sequence. Genome Res. (2005) 15:901–13. 10.1101/gr.3577405 15965027 PMC1172034

[32] Siepel A Bejerano G Pedersen JS Hinrichs AS Hou M Rosenbloom K Evolutionarily conserved elements in vertebrate, insect, worm, and yeast genomes. Genome Res. (2005) 15:1034–50. 10.1101/gr.3715005 16024819 PMC1182216

[33] Anderson GW Goebel HH Simonati A. Human pathology in NCL. Biochim Biophys Acta. (2013) 1832:1807–26. 10.1016/j.bbadis.2012.11.014 23200925

[34] Carlén B Englund E. Diagnostic value of electron microscopy in a case of juvenile neuronal ceroid lipofuscinosis. Ultrastruct Pathol. (2001) 25:285–8. 10.1080/019131201753136296 11577772

[35] Ishii M Takahashi K Hamada T Tanaka A Higami S. Cutaneous ultrastructural diagnosis of ceroid-lipofuscinosis. Br J Dermatol. (1981) 104:581–5. 10.1111/j.1365-2133.1981.tb08176.x 7236519

[36] Mole SE Anderson G Band HA Berkovic SF Cooper JD Kleine Holthaus S-M Clinical challenges and future therapeutic approaches for neuronal ceroid lipofuscinosis. Lancet Neurol. (2019) 18:107–16. 10.1016/S1474-4422(18)30368-5 30470609

[37] Inoue E Watanabe Y Xing J Kushima I Egawa J Okuda S Resequencing and association analysis of CLN8 with autism spectrum disorder in a Japanese population. PLoS One. (2015) 10:e0144624. 10.1371/journal.pone.0144624 26657971 PMC4682829

[38] Nelvagal HR Lange J Takahashi K Tarczyluk-Wells MA Cooper JD. Pathomechanisms in the neuronal ceroid lipofuscinoses. Biochim Biophys Acta Mol Basis Dis. (2020) 1866:165570. 10.1016/j.bbadis.2019.165570 31678162

[39] Tieleman DP Shrivastava IH Ulmschneider MR Sansom MS. Proline-induced hinges in transmembrane helices: possible roles in ion channel gating. Proteins. (2001) 44:63–72. 10.1002/prot.1073 11391769

[40] Sansom MS Weinstein H. Hinges, swivels and switches: the role of prolines in signalling via transmembrane *α*-helices. Trends Pharmacol Sci. (2000) 21:445–51. 10.1016/S0165-6147(00)01553-4 11121576

[41] Senes A Engel DE DeGrado WF. Folding of helical membrane proteins: the role of polar, GxxxG-like and proline motifs. Curr Opin Struct Biol. (2004) 14:465–79. 10.1016/j.sbi.2004.07.007 15313242

[42] Passantino R Cascio C Deidda I Galizzi G Russo D Spedale G Identifying protein partners of CLN8, an ER-resident protein involved in neuronal ceroid lipofuscinosis. Biochim Biophys Acta. (2013) 1833:529–40. 10.1016/j.bbamcr.2012.10.030 23142642

[43] Otsu W Kurooka T Otsuka Y Sato K Inaba M. A new class of endoplasmic reticulum export signal PhiXPhiXPhi for transmembrane proteins and its selective interaction with Sec24C. J Biol Chem. (2013) 288:18521–32. 10.1074/jbc.M112.443325 23658022 PMC3689993

