# Supplementary material for: Two compound heterozygous variants in the CLN8 gene are responsible for neuronal cereidolipofuscinoses disorder in a child: a case report
Source: Front Pediatr. 2024 May 1;12:1379254. doi: 10.3389/fped.2024.1379254 (PMC11094295; doi:10.3389/fped.2024.1379254)
Supplement: Supplementary file 1 [file Datasheet1.pdf]

Supplementary Material

1. Whole genome sequencing and bioinformatics analysis

4.696.354 variants were detected in variant calling analysis, which were later annotated and prioritized. Different sets of filters were used in order to detect potentially causative mutations:

i. Homozygous mutations in coding/splicing regions with a population frequency lower than 1%;
ii. Heterozygous mutations in coding/splicing regions with at least two variants in the same gene and a population frequency lower than 1% (compound heterozygous);
iii. Heterozygous mutations in coding/splicing regions with a population frequency less than 0,5%;
iv. Mutations found among the 73 genes considered of preventive relevance, listed by American College of Medical Genetics and Genomics (ACMG) [24].
v. Mitochondrial variants: mutations with high heteroplasmy (>10%) and in coding regions or tRNA and rRNA genes (and not part of the definition of the haplogroup), and not in D-Loop region.

Sanger sequencing was used to confirm the mutations in the index case.

2. Electron Microscopy

Tissue was embedded in resin using the standard protocol: washing in cacodylate buffer x3, postfixation in 1% osmiumtetroxide 1.5% potassium ferrocyanide for 45 min at room temperature, washing in dH20 3x for 10 min. Dehydration in upgrading alcohol solutions (30% EtOH 5 min, 50% EtOH 10 min, 70% EtOH 10 min, 95% EtOH 15 min 2x, 100% EtOH 20 min 2x) and acetone 1 h. Infiltration in upgrading acetone:araldite 1:1 30 min x1, 1:3 20 min x1, pure araldite overnight 4ºC. Tissues were placed in new pure araldite in molds and left an additional 1 h at room temperature, then polymerization at 58-60º for 48 hs. Ultrathin sections were obtained using a Leica EM UC7 Ultramicrotome. Multiple sections were visualized using an electron microscope HRSTEM JEOL JEM2100, 200kV, LaB6 with a digital camera GATAN ORIUS 1000.aterial
